# Correlating chemical diversity with taxonomic distance for discovery of natural products in myxobacteria

**DOI:** 10.1038/s41467-018-03184-1

**Published:** 2018-02-23

**Authors:** Thomas Hoffmann, Daniel Krug, Nisa Bozkurt, Srikanth Duddela, Rolf Jansen, Ronald Garcia, Klaus Gerth, Heinrich Steinmetz, Rolf Müller

**Affiliations:** 1 Helmholtz Institute for Pharmaceutical Research Saarland (HIPS), Department of Microbial Natural Products, Helmholtz Centre for Infection Research and Department of Pharmaceutical Biotechnology, Saarland University, Campus E8.1, 66123 Saarbrücken, Germany; 2 German Centre for Infection Research (DZIF), Partner Site Hannover-Braunschweig, 38124 Braunschweig, Germany; 3 Helmholtz Centre for Infection Research (HZI), Department of Microbial Drugs, 38124 Braunschweig, Germany

## Abstract

Some bacterial clades are important sources of novel bioactive natural products. Estimating the magnitude of chemical diversity available from such a resource is complicated by issues including cultivability, isolation bias and limited analytical data sets. Here we perform a systematic metabolite survey of ~2300 bacterial strains of the order *Myxococcales*, a well-established source of natural products, using mass spectrometry. Our analysis encompasses both known and previously unidentified metabolites detected under laboratory cultivation conditions, thereby enabling large-scale comparison of production profiles in relation to myxobacterial taxonomy. We find a correlation between taxonomic distance and the production of distinct secondary metabolite families, further supporting the idea that the chances of discovering novel metabolites are greater by examining strains from new genera rather than additional representatives within the same genus. In addition, we report the discovery and structure elucidation of rowithocin, a myxobacterial secondary metabolite featuring an uncommon phosphorylated polyketide scaffold.

## Abstract (web summary)

It is thought that the chances for discovery of novel natural products increase by screening rare organisms. Here the authors analyse metabolites produced by over 2300 myxobacterial strains and, indeed, find a correlation between taxonomic distance and production of distinct secondary metabolite families.

## Introduction

The search for uncharacterized medicinally relevant natural products is an important aspect of pharmaceutical research^1^. Enormous progress in genomics and metabolomics analyses of natural product-producing organisms has led to a resurgence of discovery efforts^2–4^. Success rates for finding novel scaffolds remain an important matter of debate as the re-isolation of known compounds or their derivatives is a recurring issue, especially with long-established sources like the bacteria of the actinomycetes clade^5^. Since natural product isolation is generally a laborious and often challenging endeavor, besides thorough de-replication a careful selection of environmental sources of microbes and experimental methods is required to improve the prospects of isolation procedures^6^. It is a common perception that chances for the discovery of chemical novelty increase by moving towards rarely screened organisms, such as underexplored microorganisms occupying extraordinary habitats or specific ecological niches^7,8^. Thus, a number of new natural product sources have emerged and are re-fueling the natural products supply chain, as exemplified by recent reports from marine actinomycetes, myxobacteria, and microbial plant- or sponge symbionts, just to name a few^9–11^.

In addition to new sources, the advent of high-throughput genome sequencing techniques has made accessible increasing numbers of microbial genomes for the in silico evaluation of their capacity to produce secondary metabolites^12–14^. In order to cope with this wealth of genetic information and the constantly improving understanding of natural product biosynthetic pathways, powerful tools to mine genomes for biosynthetic gene clusters (BGCs) have been developed^15–17^. While this repository of microbial BGCs has the potential to drastically improve natural product discovery, translation into comparable numbers of novel natural products has yet to be revealed. Numerous BGCs have been identified which in theory can produce unknown products. However, knowledge of genome-inscribed potential for natural products is not paralleled with the identification, isolation, and characterization of new compounds^18^. The apparent gap between the genomic capacity of a strain for secondary metabolite production (genotype) and the metabolites observed when it is cultivated (chemotype) continues to be a major bottleneck in natural product research and puts the number of theoretically available BGCs into perspective^19,20^. In line with rapidly evolving genome mining approaches, analytical methods for the in-depth characterization of natural product profiles have also seen lively development and promising application^21,22^. These improvements have been driven, at least in part, by advances in mass spectrometric instrumentation, an increasing number of databases, and inspired by metabolomics-based strategies^23–26^. These workflows serve as the complementary tool set to compare and contrast genomics-derived expectations with the reality of secondary metabolite production^27,28^.

In this study, we provide a metabolite-centric view of observable natural product diversity within the myxobacteria, an important group of secondary metabolite producers^29,30^. We deliberately focus on natural products that are detected under laboratory cultivation conditions as this is the typical scenario of natural product discovery and isolation programs. This workflow is generally utilized due to its practical feasibility, scalability, and biotechnological manageability required for further downstream compound development. Continued efforts to characterize myxobacterial taxa have culminated in the description of 10 families, 30 genera, and 62 species spread over 3 suborders^31–33^. These numbers are still strikingly below those documented for other well-known secondary metabolite-producing clades, i.e., the actinomycetes (with over 2500 validly described species^34^). Therefore, we reason that within the myxobacteria phylogenetic group chances to identify new taxonomic branches are plentiful and each discovered strain may afford new chemical entities. Achievements in natural product isolation from myxobacteria suggest that taxonomy is, to some extent, represented by genus-typical secondary metabolites. In contrast to recently published work focusing on large numbers of producer strains analyzed for biosynthetic gene cluster information^20,35^, we set out to exclusively use LC–MS data sets. A total of ~2300 myxobacterial extracts was the basis to establish statistical evidence proving the degree of taxonomic diversity is linked to measurable secondary metabolite diversity which, in turn, corroborates our efforts to continue uncovering new myxobacterial genera and their unprecedented natural products.

## Results

### Exploring the order *Myxococcales*

**Fig. 1:**
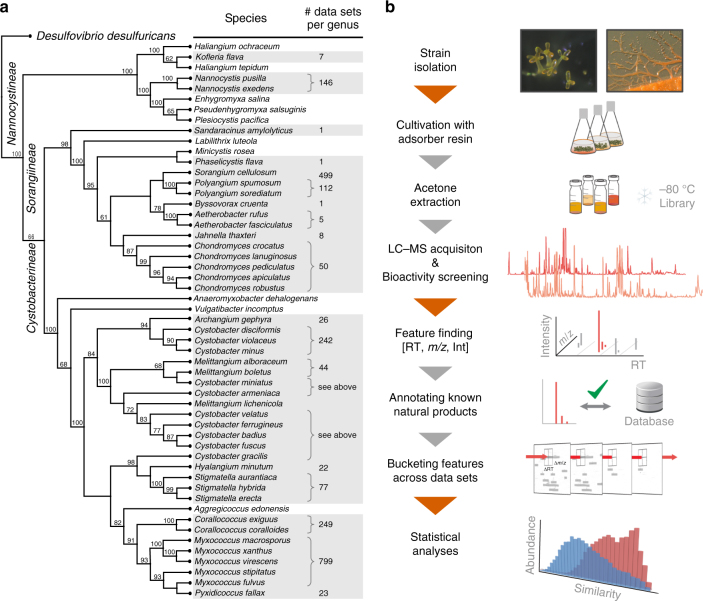
Myxobacterial metabolite profile analysis. **a** Taxonomy of the order *Myxococcales* based on a neighbor-joining consensus tree constructed from 16S rRNA gene sequence data of type strains, with the number of available LC–MS data sets given for each genus. Additional strains not assigned to the listed species were included for some genera, for a total count of 2316 data sets (Supplementary Table 8 and Supplementary Data 1). **b** Outline of the analysis workflow used in this study

Myxobacteria are typically soil-living, Gram-negative proteobacteria that are found in most climate zones around the globe^32^. These slow-growing bacteria impress with abilities such as swarming, formation of multicellular fruiting bodies, and the production of a large repertoire of secondary metabolites^10,36^. In the 1980s, research groups of Reichenbach and Höfle initiated the collection of myxobacterial strains and secondary metabolites while working at the HZI (formerly GBF, Braunschweig, Germany) which has now cumulated in more than 9000 strains. Of those, a subset of ~2300 strains has been selected for analysis in a standardized fashion in the course of an ongoing screening campaign. The strains used for this project were chosen to achieve high coverage in myxobacterial taxonomy (Fig. 1a). All strains were cultivated in empirically optimized, genus-typical cultivation media (Supplementary Note 3, Supplementary Tables 6 and 7) and processed according to standardized protocols for extract preparation, LC–MS measurement, data analysis, and de-replication, thereby setting the stage for a large-scale comparative analysis (Fig. 1b).

### Distribution of known myxobacterial compounds

**Fig. 2:**
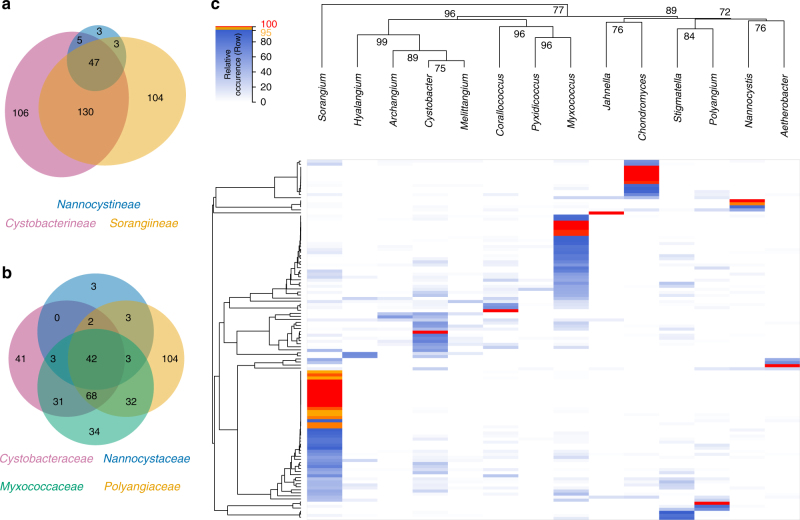
Distribution of known myxobacterial compounds amongst extracts from ~2300 strains. **a** Venn diagram on suborder level. **b** Euler plot on family level (overlap numbers between opposite circles not printed). **c** Heatmap displaying known compound families found among the myxobacterial genera after UPGMA-based hierarchical clustering. Red color indicates 100% of detections within one genus, i.e., the compound family is solely found in this genus although not necessarily in all data sets of the respective genus. Compound families that were more than 95% occuring within one genus are orange. The data sets originate from genera comprising at least 5 corresponding strains: *Aetherobacter* (5 strains), *Archangium* (26), *Chondromyces* (50), *Corallococcus* (249), *Cystobacter* (242), *Hyalangium* (22), *Jahnella* (8), *Melittangium* (44), *Myxococcus* (799), *Nannocystis* (146), *Polyangium* (112), *Pyxidicoccus* (23), *Sorangium* (499), and *Stigmatella* (77). A total of 170 metabolite families were considered, covering 398 compounds. Column clustering was subjected to bootstrapping (*n* = 500) with ‘approximately unbiased’ *p*-values displayed

At the time of data processing, our in-house database contained 170 structurally characterized myxobacterial metabolite families comprising 398 compounds. LC–MS data sets from a total of ~2300 extracts were examined for these compounds based on accurate *m*/*z*, retention time, and isotope pattern fits to reference data obtained from the respective purified compounds. A compound distribution matrix comparing compound family to taxonomic diversity was created as a result of this compound annotation procedure. Individual annotation results from all data sets were a priori grouped according to the suborder, family, or genus and processed to yield taxonomic distributions. Following this approach, the taxonomic position of every data set was known and used for clustering. The distributions for suborder and family are illustrated using Euler diagrams (Fig. 2a, b) and a heatmap is shown for the genus level (Fig. 2c).

**Fig. 3:**
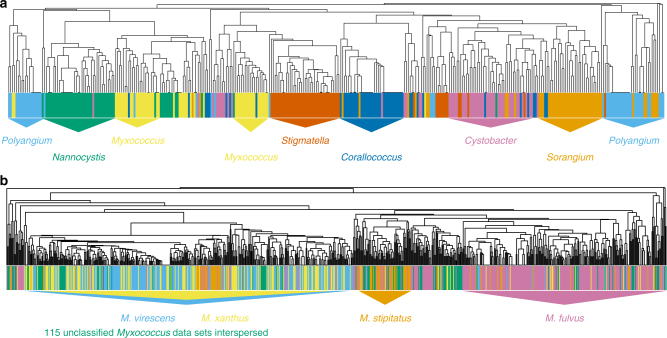
Metabolite profiles clustering by taxonomy. **a** Clustering of 350 data sets based on the distribution of identified known myxobacterial compounds. Each of the 7 most abundant myxobacterial genera is represented by 50 randomly selected data sets with a distinguishable color coding. Colored triangles indicate regions which are enriched with data sets of a single genus. A total of 94 compound families was found amongst these data sets. **b** Clustering of all available *Myxococcus* data sets (790) covering a varying number of representatives of *M. virescens* (189, blue), *M. xanthus* (154, yellow), *M. stipitatus* (76, orange), *M. fulvus* (256, magenta), and unclassified *Myxococcus* species (115, green). See Supplementary Figures 6 and 7 for additional considerations

While a number of compounds are distributed in several genera, a striking subset exists that is either unique or highly specific to a certain genus. Compound families which are unique to a genus are highlighted in red while compound families exhibiting high genus-specificity are colored in orange (Fig. 2c). The pattern observed from this approach provided strong evidence for the existence of distinct chemotypes. Since the available data sets do not uniformly represent the different genera in terms of sample numbers, the heatmap representation requires careful interpretation. For instance, in our analysis only five *Aetherobacter* sp. represent the genus *Aetherobacter*^37^, while other genera like *Sorangium* have several hundred representatives. Thus, it is safe to conclude that the currently known *Aetherobacter*-specific compounds are not produced by *Sorangium* species. In contrast, concluding that a typical *Sorangium*-specific compound is never produced by *Aetherobacter* is not valid as the sample size of *Aetherobacter* data sets is presently too low. Nonetheless, there is notable genus specificity in myxobacterial natural product distributions, and it is an important aspect of this analysis that genus-specific compounds readily were detected under common laboratory cultivation conditions. This finding, which we term ‘taxonomy paradigm, supports the idea of preferentially exploring new genera to increase the likelihood of finding novel natural product scaffolds. The recently characterized jahnellamides and aetheramides provide examples of this idea^38,39^. In our heatmap, these two compound classes are represented by the red colored areas of *Jahnella* and *Aetherobacter*, respectively. It was more difficult to use our LC–MS data set to stringently define taxonomic groups of species (see below, Fig. 3b) and thus we refrained from conducting the analogous analysis below genus level.

### Clustering individual data sets

Along those lines we asked two additional questions: (1) Will LC–MS data sets self-organize to genus-related clades based upon the genus they belong to? and (2) Will data sets also cluster on species level? To determine whether Myxobacteria would self-organize into genus and species based upon only chemotype, the analysis was repeated with individual, blinded data sets. We randomly selected 50 data sets from each of the seven most frequent genera in our collection and subjected the individual files to hierarchical clustering. We found that clustering the data based on known metabolites only was sufficient to allocate most of the data sets into genus-featuring clades (Fig. 3a). This result further suggests that myxobacterial genera have significant inter-genera variations of their secondary metabolome.

Whether, and to what extent, a sub-genus level clustering is feasible was addressed by analyzing 790 data sets from four different *Myxococcus* species including unclassified *Myxococcus* species. Conducting this enlarged sample set also revealed a general tendency toward species-typical compounds for the genus *Myxococcus* (Fig. 3b). However, this tendency was much less clear than on genus level as seen for the essentially not distinguishable species pair *M. virescens/M. xanthus* in (Fig. 3b), whereas *M. fulvus* and *M. stipitatus* were separated from the other *Myxococcus* species. While we can rule out spatial or temporal bias during isolation of this strain set, taxonomic misclassification on species level cannot be excluded completely and may contribute to incomplete species separation. On the other hand, numbers of known metabolites from *Myxococcus* are still limited and may not be sufficient yet to allocate all species in separate clusters.

### A view on unknowns

**Fig. 4:**
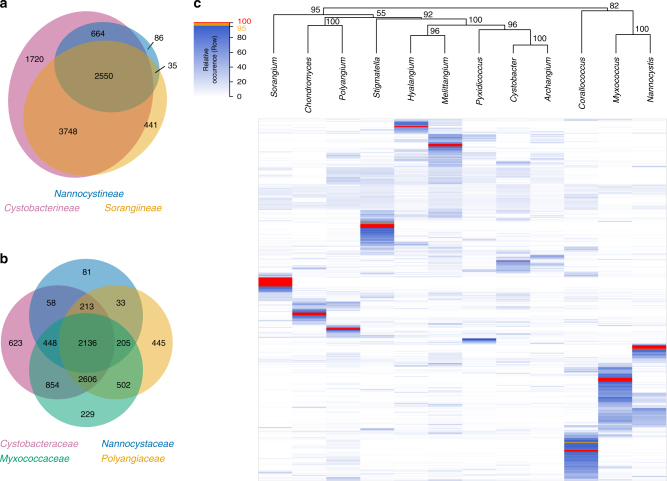
Clustering of unknown features. Distribution of mass spectral buckets created from unknown features of 515 myxobacterial strain extracts shown as **a** Venn diagram on suborder level and **b** Euler plot on family level. **c** Heat map displaying ~9200 buckets across all data sets. The data sets are distributed as follows: *Archangium* (26), *Chondromyces* (50), *Corallococcus* (50), *Cystobacter* (50), *Hyalangium* (22), *Melittangium* (44), *Myxococcus* (50), *Nannocystis* (50), *Polyangium* (50), *Pyxidicoccus* (23), *Sorangium* (50), and *Stigmatella* (50). Individual data sets were collapsed to genus level before applying UPGMA-based hierarchical clustering. Buckets unique to a genus are shown in red, those which are highly specific with >95% relative occurrence per genus are in orange (visible upon magnification). Column clustering was subjected to bootstrapping (*n* = 500) with ‘approximately unbiased’ *p*-values displayed. Contains no technical or biological replicates

Even though the aforementioned analyses allowed definition of genus-specific secondary metabolites, it was solely based on several hundred previously characterized compounds representing basically all known myxobacterial natural products (the ‘knowns’). Thus, we set out to apply an unbiased method for additional analysis that uses all mass spectrometric features from data sets (represented by *m*/*z*, retention time, isotope pattern) including initially all knowns and unknowns rather than only the restricted set of knowns. It should be noted that any natural product can be responsible for numerous mass spectrometric features, i.e., a single chemical entity may result in different ion adducts alongside with in-source-generated fragment ions. All of those will create independent features contributing to the complexity of each data set. For the following analysis we selected 515 data sets from 12 myxobacterial genera with a maximum of 50 data sets for each genus. We used all available data sets for the genera with <50 representatives whereas data sets were randomly selected if >50 were available. Genera with <20 representatives were not included in this analysis. Following the annotation of mass spectral features, all data sets were filtered to remove typical polymer impurities from the extraction process and cultivation media-related features (‘background’ features). Filtering background features was an essential step for this type of analysis as we intended to establish downstream analyses based on features specific to bacterial growth and at the same time ignore media components. Finally, ~220,000 features were merged into ~9200 buckets with a bucket being defined as an *m*/*z* and RT region hosting all features with matching *m*/*z* and RT. The occurrence of buckets across all data sets was realized by means of a distribution matrix. The matrix may be transformed to represent different taxonomic levels by merging individual data sets into groups, e.g., to obtain the distribution on genus level which led to the results shown in Fig. 4.

Intriguingly, bucket distributions are highly different for the various genera with buckets being even unique or largely specific to a single genus as indicated by the red areas and orange lines in Fig. 4c. Some slightly blueish colored areas extend over many genera while densely colored areas indicate relative occurrences specifically enriched within certain genera. The influence of the different cultivation media on clustering had demonstrably low impact on the overall result as background-features were disregarded in this approach (Supplementary Note 2 and Supplementary Figures 7, 9). For instance, *Chondromyces* and *Polyangium* were grown in the same medium but showed an obvious separation in the heatmap. As a consequence, we conclude that the observed clustering is predominantly caused by strain-typical chemical entities including known and unknown natural products. The heatmap approach thus has the potential to highlight putative novel metabolites when used as part of a screening campaign, as metabolomic data for new strains added to the collection can be channeled readily into the analysis framework.

**Fig. 5:**
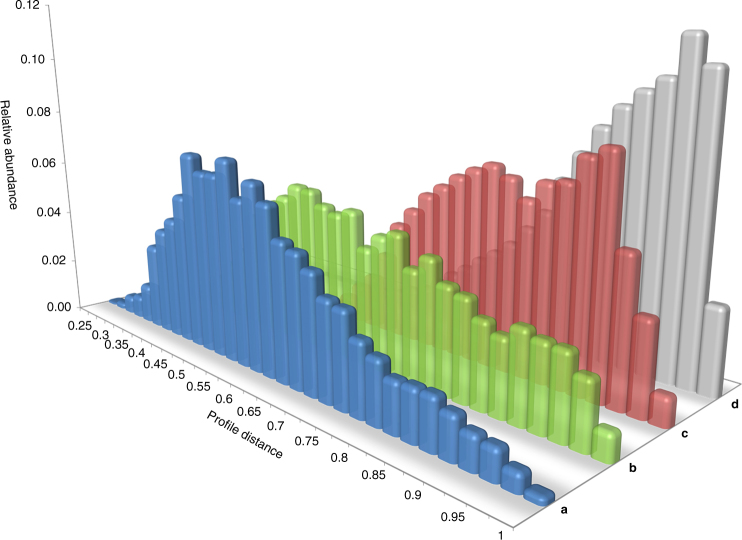
Myxobacterial metabolite profile similarity in relation to taxonomic distance. Histogram plots depicting binned distributions for metabolite profile (dis)similarity within and between varying taxonomic ranks, whereas on *x* axis scale 0 means highest similarity, 1 means greatest distance. Distance distribution calculated between all profiles from: **a** – various strains within their respective species (blue), **b** – strains belonging to different species but within their respective genus (green), **c** – strains belonging to different genera but within their respective family (red), **d** – strains belonging to different suborders (gray). See Supplementary Table 5 for readouts from statistical tests

Having metabolite distributions for the (approximately taxonomically balanced) set of 515 strains spanning 12 genera and ~9200 bucketed features in hand, we set out to explore the (dis)similarity of metabolite profiles as a function of taxonomic relatedness. We were interested whether a meaningful correlation between taxonomic distance and observed chemotype diversity could be substantiated through statistical analysis. For that purpose the occurrence pattern of buckets per strain were converted into a matrix comprising 515 binary profiles and pairwise distance was determined using the bitvector cosine distance measure. Clearly, between-clade metabolite profile distances are on average larger than distance values calculated internally within members of the respective clade, as is evident from distribution plots and hypothesis testing (Fig. 5; paired *t*-test, *p*<<0.001; Supplementary Note 2, Supplementary Figure 10 and Supplementary Table 5). The effect is somewhat moderate on species level, but much more pronounced on genus level (Fig. 5a versus 5b, c, d).

The outcome of this taxonomy-resolved profile similarity analysis has an important strategic implication for strain isolation efforts: chances to encounter new bacteria exhibiting a markedly different metabolite profile are clearly increasing when samples belonging to new species, genera, families and suborders are analyzed as opposed to extending the sheer number of strains within a given species. The positive correlation between taxonomic distance and chemotype diversification confirmed here translates into considerably improved prospects for the discovery of metabolites not contained in previously available samples. Expectations for novel compound discoveries rise particularly when traversing beyond species level to include isolates from additional genera (Fig. 5, green vs. red series).

### A new natural product from *Sorangium cellulosum*

**Fig. 6:**
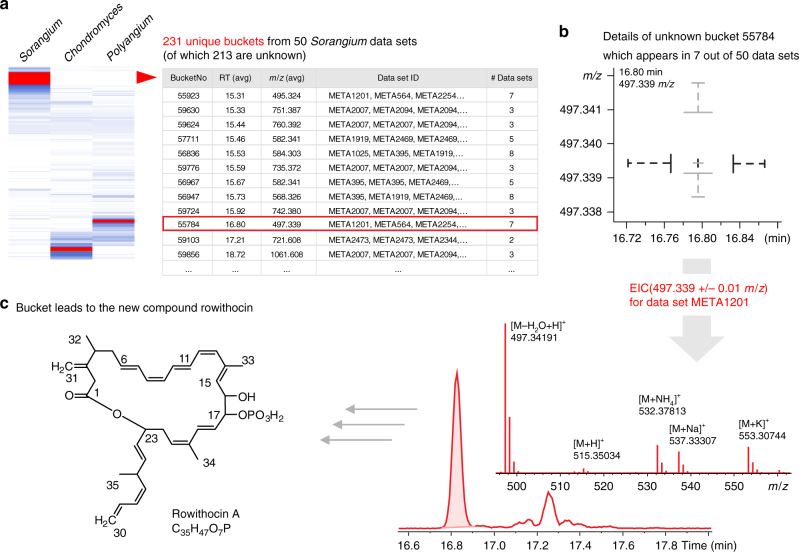
Discovery of the new myxobacterial natural product rowithocin. **a** Section of the heatmap featuring unique buckets from *Sorangium*. More than 200 buckets are found exclusively within the 50 *Sorangium* data sets. **b** Information on each bucket allows interpretation of the bucket’s characteristics such as median, quantile and outlier analysis. **c** Investigation of an unknown bucket with 497.339 *m*/*z* around 16.8 min revealed that the bucket corresponds—besides several adduct ions—to a distinct signal of type [M + H]^+^ with 515.350 *m*/*z*. Subsequent analyses of newly prepared extracts revealed additional related compounds with significantly increased abundance which were eventually characterized as the novel myxobacterial metabolite family of rowithocins. Rowithocin A (C_35_H_47_O_7_P) is the largest derivative with a monoisotopic mass of 610.3059 Da, detectable in fresh extracts as [M + H]^+^ (611.3132 *m*/*z*) or [M – H]^−^ (609.2986 *m*/*z*)

The above survey of unknowns has demonstrated how taxonomic analysis of myxobacterial metabolite profiles may assist to nominate new natural product candidates on the basis of their predominant (or even exclusive) appearance within specific taxonomic clades. We thus sought to exploit the capability of this approach to guide the discovery and identification of a hitherto uncharacterized compound. Even for *Sorangium*—arguably the best studied myxobacterial genus—many clade-specific buckets highlighted by the clustering procedure remained unidentified after querying them against our compound database. Due to correlation to anti-staphylococcal bioactivity, a single late-eluting bucket at 16.80 min with 497.339 *m*/*z* was selected for a more detailed examination (Fig. 6a). The best producer strain, *S. cellulosum* MSr2476, was selected for further investigation and showed a distinct peak at the expected retention time plus smaller additional peaks of isobaric derivatives. The spectra comprising the peak revealed that 497.339 *m*/*z* resulted from a strong in-source fragmentation of an unknown compound with an [M + H]^+^ of 515.350 *m*/*z* (Fig. 6b, c).

Identification of additional compound family members was inferred from characteristic UV absorption and tandem mass spectrometry (MS/MS) fragmentation patterns using fresh XAD-based extract generated from *S. cellulosum* MSr2476. Amongst those, the most abundant signal was isolated and the structure elucidation yielded the new compound rowithocin A (Supplementary Note 1, Supplementary Figures 1–4, Supplementary Tables 1–4), a previously uncharacterized and rather instable natural product belonging to the phosphorylated polyketide class of antibiotics^40^. The bucket of 497.339 *m*/*z* which initiated the identification of rowithocins is a moderately stable, dephosphorylated elimination product, which endured the long process each extract underwent in the course of this project. This example shows that the data analysis workflow utilized here is capable to pinpoint real and novel natural products. The fact that all analyzed genera feature numerous unique buckets is a striking result that clearly strengthens the taxonomy paradigm in natural product discovery.

## Discussion

Microbial secondary metabolites continue to be valuable and, inarguably, essential sources to furnish the pharmaceutical drug discovery pipelines, and the panel of methods for their discovery has been amended recently by genomics-guided approaches^2,17^. However, DNA sequencing and in silico analysis revealing ‘theoretical compound production ability’ currently lacks straight forward technology to translate novel biosynthetic gene clusters into truly new metabolites at larger scale. As a consequence of the present study we propose metabolomics-based chemo-taxonomic distribution analyses as a complementary tool to guide the identification of chemically detectable new natural products without any requirement for genomic knowledge. Nevertheless, it should be mentioned that the relatively few currently available myxobacterial genomes demonstrate—as is the case for other microbial producers—enormous potential for the production of presently unknown metabolites^29,41^. Provided that the strain collection under investigation exhibits adequate taxonomical diversity and coverage, the ‘compounds first’ approach taken here has the potential to directly pinpoint candidates for novel metabolites with a high implicit likelihood for structural novelty. This conclusion is supported by the correlation between the degree of metabolome diversification and taxonomic distance substantiated in this study. We argue that taxonomic distribution analysis of candidate signals can be especially useful to support the discovery of new natural products when it is combined with additional rationales. In that sense, a ‘double filtering’ approach was applied for rowithocin discovery in this study: bioactivity data suggested the presence of a compound of potential interest within a specific retention time window in *Sorangium* extracts which, however, left numerous unknown *m*/*z* candidates that could be responsible for the observed biological activity (as is often the case in the course of bioactivity guided fractionation). Taxonomic distribution analysis in this case can help to narrow down the list of candidate features by their likelihood to represent a new taxa-specific metabolite.

For myxobacteria in particular, we show that metabolite profiles display considerable genus specificity and that chances for novel compound discovery are markedly increasing when profile-based analysis is extended to include members of taxonomically distant clades. These findings also have implications for the strategic focusing of screening programs on underexploited taxa, suggesting intensifying efforts for both the re-evaluation of known representatives and the isolation of new members. In the case of (probably not only) myxobacteria—featuring to date relatively few described clades—novel genera and families should be brought into culture and preferentially analyzed in depth to support drug discovery programs.

## Methods

### Sample cultivation and extract preparation

Strains were grown in 250 mL shaking flasks with 100 mL of a genus-typical medium supplemented with 2% (v/v) of sterile, aqueous adsorber resin suspension (XAD-16, Rohm & Haas). Aeration of the cultures was guaranteed by using non-sealing aluminum caps. The flasks were inoculated with 10 mL of a starter culture and incubated for 10 to 14 days at 30 °C. After incubation, cells and XAD were removed from the broth using a 100 µm metal sieve, washed with DI water, and subsequently extracted in glass Erlenmeyer flasks with 35 mL of acetone under constant shaking. The organic extract was filtered, evaporated to dryness, and suspended in 1.1 mL of methanol utilizing an ultrasonic bath to eventually obtain the crude extract. Crude extracts were transferred to polypropylene tubes and stored at −80 °C until measurement. Extracts were prepared and collected over several years starting from 2000 to 2014. Prior to LC–MS analysis, samples were allowed to return to room temperature before being centrifuged for 3 min with at least 10,000 × *g*.

### LC–MS analysis

All measurements were performed with a Dionex Ultimate 3000 RSLC system (Thermo) using a BEH C18, 100 × 2.1 mm, 1.7 µm *d*_p_ column (Waters). Separation of 1 µL sample was achieved by a linear gradient from (A) H_2_O + 0.1% FA to (B) ACN + 0.1% FA at a flow rate of 600 µL/min and 45 °C. The gradient was initiated by a 0.5 min isocratic step at 5% B, followed by an increase to 95% B in 18 min to end with a 2 min step at 95% B before reequilibration with initial conditions. UV spectra were recorded by a DAD in the range from 200 to 600 nm. The LC flow was split to 75 µL/min before entering the maXis 4 G q-ToF mass spectrometer (Bruker Daltonics) using the Apollo II ESI source. The split was set up with fused silica capillaries of 75 and 100 µm I.D. and a low dead volume tee junction (Upchurch). Mass spectra were acquired from 150–2500 *m*/*z* at 2 Hz scan rate in centroid mode. Each run started with a calibrant peak of basic sodium formate solution which was provided by a filled 20 µL loop switched into the LC flow at the beginning of each run. All measurements were performed within 17 months with a single 96-well plate measured at a time. Performance and operation of the system was thoroughly monitored to assure best possible retention time stability and *m*/*z* accuracy. A total of 346 myxobacterial compounds from our in-house isolation efforts were measured on the same system to obtain reference retention times as well as the information on typically observed ion types.

### Raw data processing

All raw data files were processed using scripted commands in DataAnalysis 4.2 b383 and TargetAnalysis 1.3 b383 (Bruker Daltonics). Files were initially calibrated with the internal calibrant peak using a polynomial fit function (HPC) for the main sodium formate cluster peaks in the range from 150 to 1400 *m*/*z*. The average *m*/*z* error from 2500 calibrations is 0.59 +/− 0.25 p.p.m. and never worse than 2 p.p.m. for the calibrant signals. Lock mass calibration was not necessary as the system remained stable over long periods. Mass spectrometric features were annotated in the range from 0.5 to 20.5 min with the proprietary FindMolecularFeatures (FMF) algorithm (Bruker Daltonics). FMF settings were as follows: S/N threshold = 2, correlation coefficient threshold = 0.7, minimum compound length = 10 spectra, smoothing width = 3 and additional smoothing activated. Adduct ion annotation was not activated to avoid downstream effects by false annotations. The feature list of each data set was exported in xml file format for subsequent import to a database (see ‘Data management’).

### Data management

An in-house SQL database system has been established to handle all data related to myxobacterial research, the Myxobase. One of many functions is the storage of and access to strain-related LC–MS data including all features. Processing results are imported to the database in a fully accessible format and automatically linked to the respective data sets they originate from. All compounds, strains, and individual data sets and even mass spectral features have unique identifiers for easy access and cross-linkage of information. The open platform data handling software KNIME 3.1.2 was used throughout the project to handle and modify the large complex data tables which were directly retrieved from the Myxobase SQL backend^42^.

### De-replication workflow for known compounds

Raw data sets were screened for known myxobacterial compounds using TargetAnalysis (TA) software (Bruker Daltonics). Identification is based on retention time, accurate *m*/*z*, and isotope pattern matching to a list of 346 myxobacterial compounds belonging to 118 compound families. Hits need to have less than 5 p.p.m. *m*/*z* error, a retention time deviation not exceeding +/− 0.15 min and an isotope pattern fit of <50 mSigma (Bruker proprietary unit; the less the better). For compound annotation we exclusively looked for the individually observed ion adduct types for each of the known compounds. Results were imported into the Myxobase database and therein linked to the individual data sets. This enables calculating distribution matrices for all compounds across all data sets.

### Data mining workflow for unknown compounds

Background filtering: the feature data sets had to be filtered prior to bucketing in order to remove features that are related to bacterial growth. This included especially cultivation medium-derived features and features related to polymer impurities of the XAD-16 adsorber resin as experimentally verified. Fortunately the latter impurities are of low intensity and thus removable without affecting too many other features. As a workaround typical polymer-related impurities were removed from each data set by applying two filters between 9 and 13 min where (1) in the range from 500–800 *m*/*z* all features with Int <20,000 were filtered and (2) in the ranges 150–500 and 800–1,600 *m*/*z* all features with Int < 5000 were removed. The reasonable intensity range for the mass spectrometer in use is between 2000 and 1.25 × 10^6^ counts. The filtering procedure can however not account for ion suppression effects possibly caused by the polymer impurities.

The second filtering step intends to remove cultivation medium-related features. Consequently, extracts were grouped according to the cultivation media that were used for fermentation (Supplementary Note 3 and Supplementary Table 7). The MS features derived from five different blank media extracts were used as reference for filtering. Media extracts were combined in different groups to best represent the media that have been used for cultivation. Subsequently, every feature data set was filtered for background features. Features were removed from a data set whenever they would fall in a 0.2 min and 0.03 *m*/*z* window of a background feature, resulting in an average feature reduction of 20%.

Bucketing: Filtered features were subjected to a bucketing process. Rectangular buckets of 0.2 min and 0.03 *m*/*z* were created and the features of all data sets were assigned to a bucket whenever retention time and *m*/*z* value matched. The final retention time and *m*/*z* value of each bucket is the average of its features. Buckets were subsequently subjected to a database search to annotate buckets that belong to known myxobacterial compounds as inferred from retention time and *m*/*z* matches. Following this approach, a distribution matrix across all features and all data sets was achieved with individual features being represented as bucket.

Clustering: The distribution matrix was collapsed from single data set level to species or genus level by combining all data sets that belong to a distinct species or genus, respectively, and counting the occurrences. Those distribution matrices served as input for a hierarchical clustering in R (hclust and pvclust) to eventually create the heatmaps shown herein using the heatmap.2 package in R^43^. With the imbalance of strain counts within genera comes the risk to invoke the impression that some genera are much more potent than others in terms of numbers of specific metabolites, which we consider misleading as it could be attributed at least in part to the significantly different numbers of investigated strains in the various genera. We therefore based our analysis of unknowns on a more balanced subset (though not a fully equilibrated subset) of data sets derived by random subsampling.

Profile similarity analysis: The distribution of MS-derived buckets across strains was converted into a binary matrix and analyzed using modules from the KNIME software package^42^. Profile similarity was calculated using the ‘Bitvector Cosine’ distance function. Means of similarity distributions were compared using paired *t*-tests with a 99% confidence interval.

### Code availability

Custom computer code developed for processing is available at Bitbucket (https://bitbucket.org/hipsprojects/).

### Data availability

Mass spectrometry data sets analyzed in this study have been deposited in the MassIVE repository under the accession code MSV000081861. Additional electronic documents, containing analysis workflows and results, are freely accessible at Bitbucket (https://bitbucket.org/hipsprojects/). The authors declare that other relevant data supporting the findings of the study are available in this article and its Supplementary Information files, or from the corresponding author upon request.

## Electronic supplementary material

Supplementary Information

Description of Additional Supplementary Files

Supplementary Data 1
