# Supplementary material for: Correlating chemical diversity with taxonomic distance for discovery of natural products in myxobacteria
Source: Nat Commun. 2018 Feb 23;9:803. doi: 10.1038/s41467-018-03184-1 (PMC5824889; doi:10.1038/s41467-018-03184-1)
Supplement: Supplementary file 1 — Supplementary Information [file 41467_2018_3184_MOESM1_ESM.pdf]

Supplementary Information

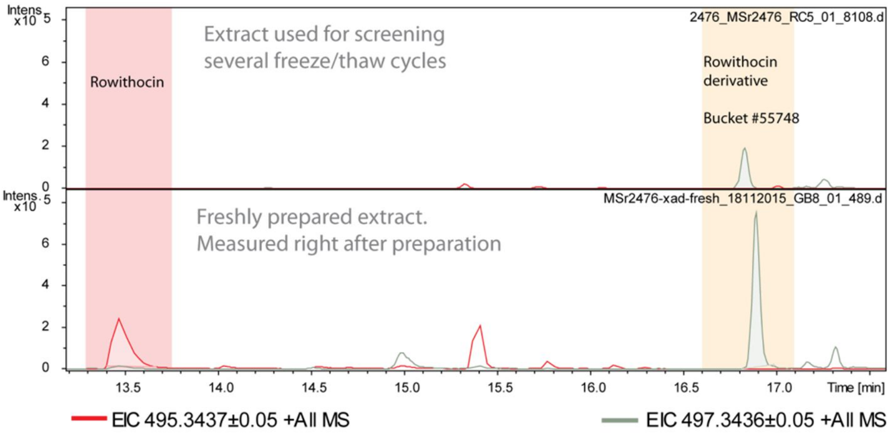

Supplementary Fig. 1: *Extracted ion chromatograms for 497.3437 and 495.3436 m/z for the extract that was used in the screening project and a freshly prepared extract of the same strain. The rowithocin peak at 13.5 min is only seen in the fresh extract.*

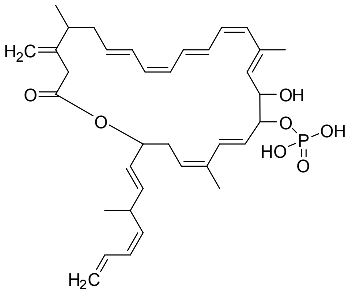

Chemical Formula: $C_{35}H_{47}O_7P$
Exact Mass: 610.30594

Supplementary Fig. 2: *Structure of rowithocin (**1**).*

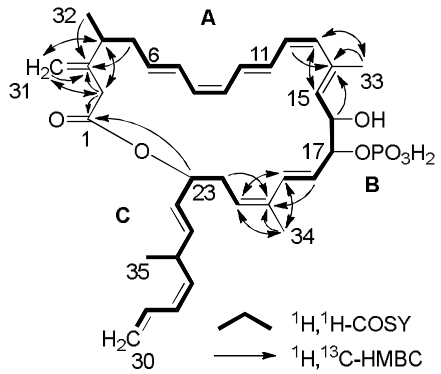

Supplementary Fig. 3: *COSY Sequences A, B, and C and selected HMBC correlations of rowithocin (1).*

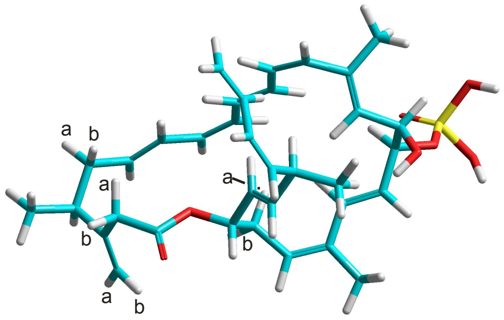

Supplementary Fig. 4: *Pm3-calculated model of rowithocin (1).*

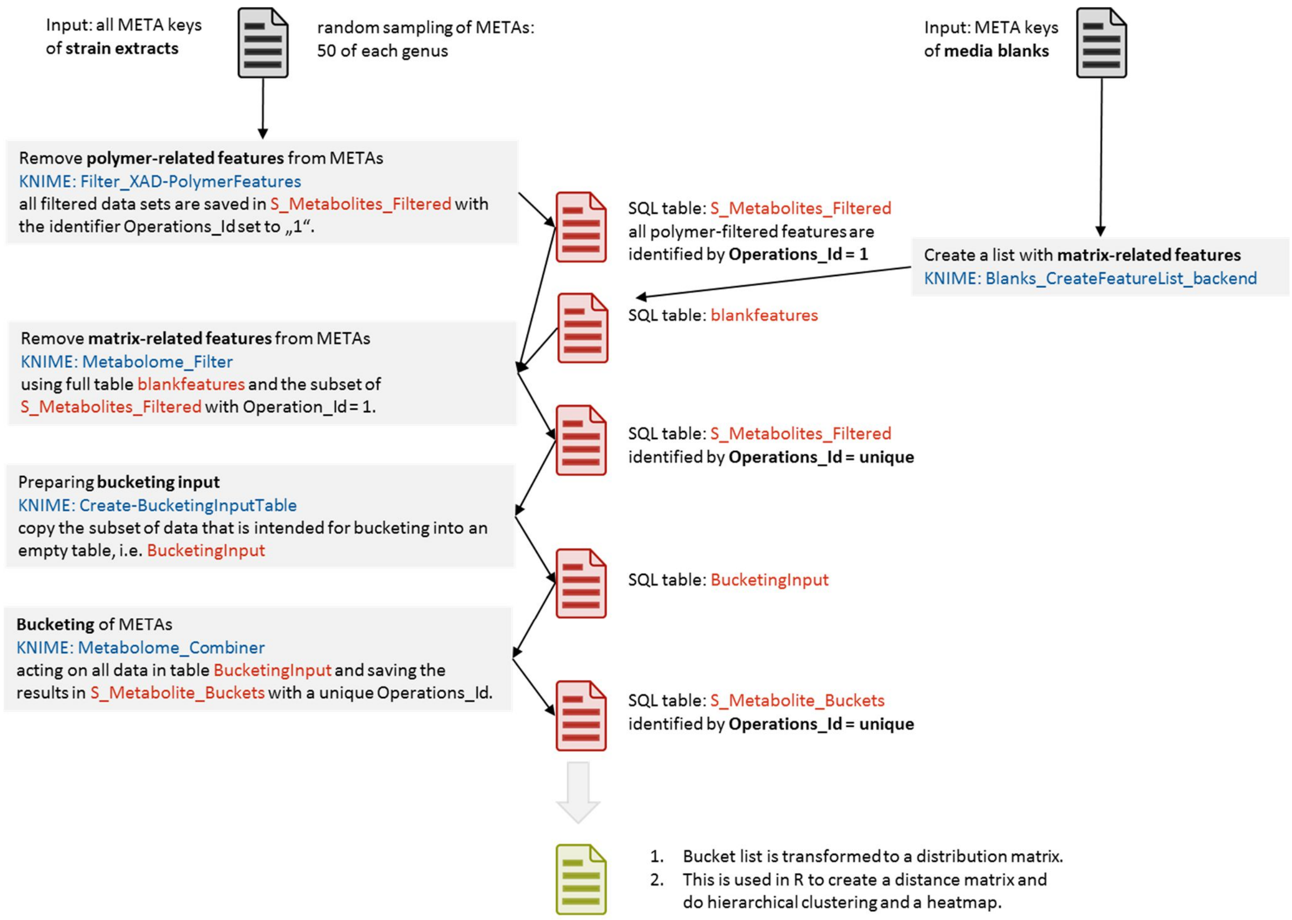

Supplementary Fig. 5: *Diagram of the general data flow for the MS feature analysis.*

10 of each genus

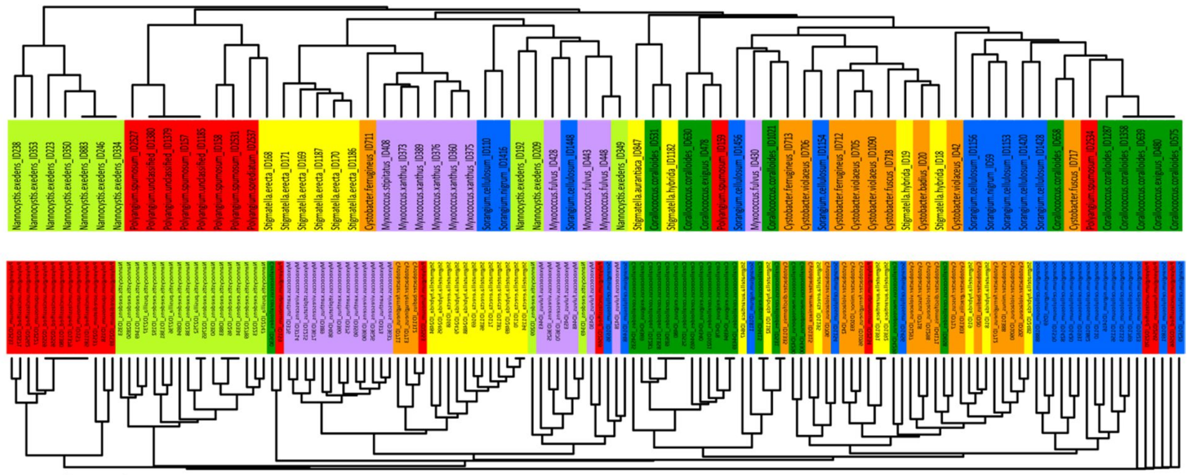

20 of each genus

Supplementary Fig. 6: *Clustering based on the occurrence of known and identified secondary metabolites with different sample set size. Reducing the sample set size to 50 % still resulted in a grouping of the different genera. Genera are color-coded.*

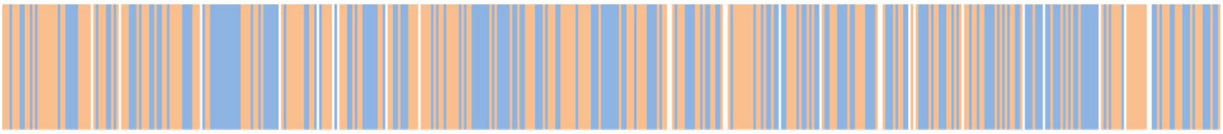

Supplementary Fig. 7: *All 499 Sorangium data sets were used for a hierarchical clustering based on 7000 buckets created from mass spectral features. The resulting order after clustering is displayed while all H medium extracts were colored in orange and all S medium extracts in blue. In case the medium was different from H and S, the bar remained white. A clustering by cultivation medium is not seen.*

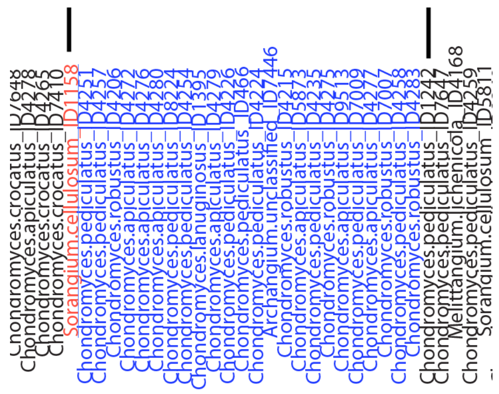

Supplementary Fig. 8: *All the blue Chondromyces belong to the apicularen-producing clade. The clade boarders are indicated with the two bars shown on top. A single S. cellulosum is located at the boarder of the clade which is sound as this S. cellulosum is indeed an apicularen producer (DistMatrix10_50-of-each_named.csv based on file SCRN_id-List04_50-of-all.csv).*

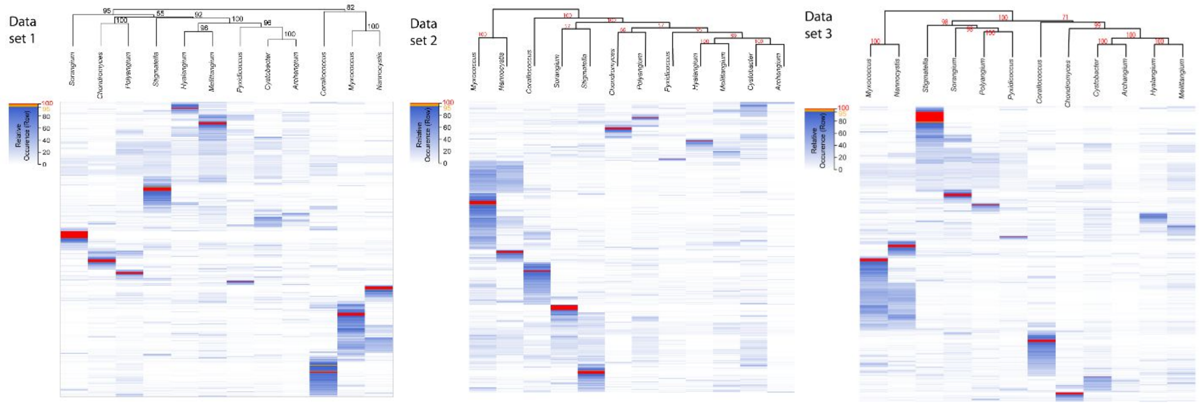

Supplementary Fig. 9: *Heat maps displaying the distribution of ~9,200 buckets across 515 random-sampled datasets from 12 myxobacterial genera. Dataset 1, as shown in the main text Figure 4; Dataset 2 and 3, using alternative randomly-sampled datasets with 515 representatives.*

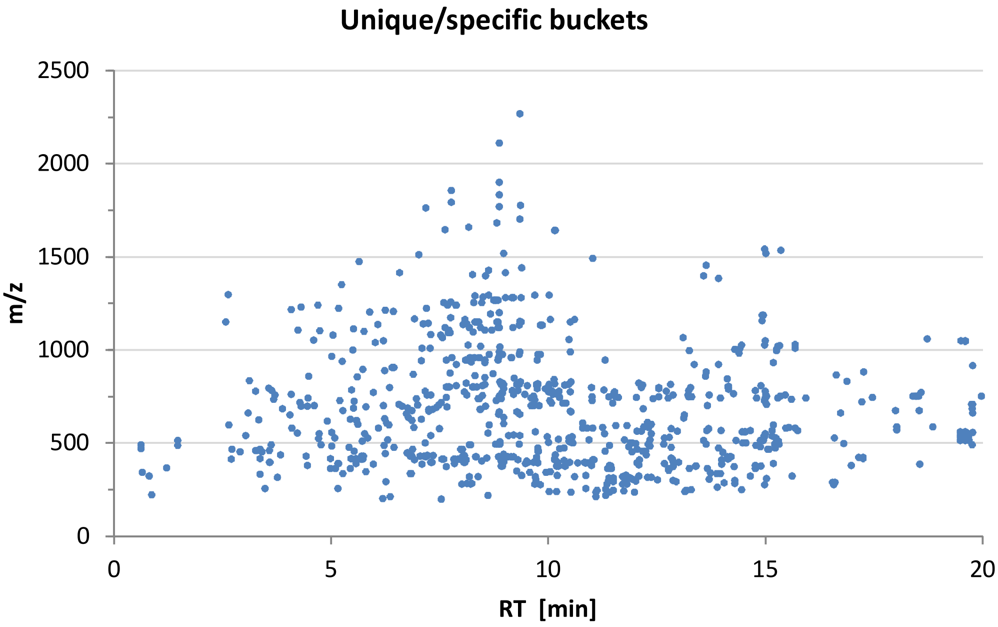

Supplementary Fig. 10: *Distribution of unique/specific buckets revealed by clustering analysis shown as 2D-scatterplot. RT, retention time in minutes; m/z, mass to charge ratio.*

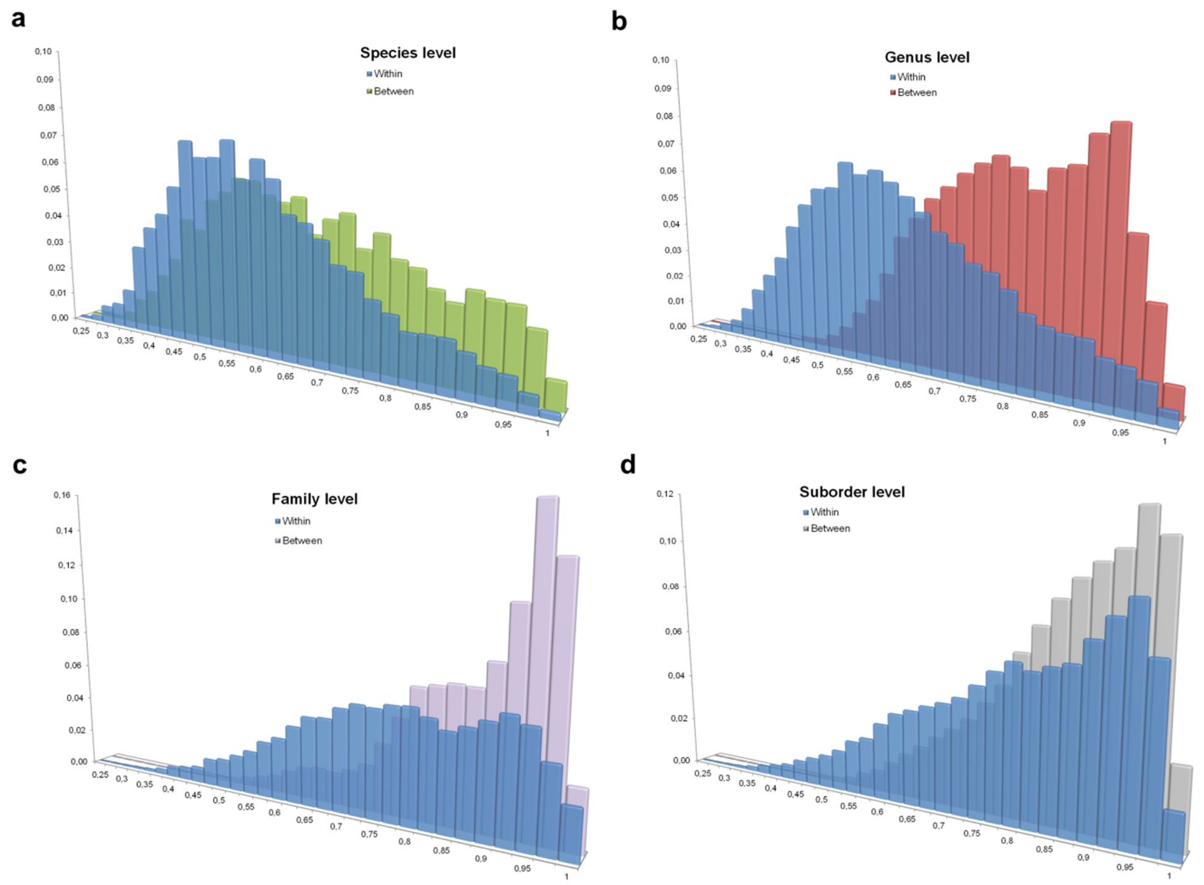

Supplementary Fig. 11: *Histogram plots depicting binned distributions for metabolite profile (dis)similarity (x axis scale: 0 = highest similarity, 1 = greatest distance) within and between varying taxonomic ranks. Distance distribution calculated between all profiles from: a - various strains within their respective species (blue) and strains belonging to different species but within their respective genus (green), b – various strains within their respective genus (blue) and strains belonging to different genera but within their respective family (red), d – various strains within their respective family (blue) and strains belonging to different families but within their respective suborder (red), d – various strains within their respective suborders and strains belonging to different suborders (grey).*

Supplementary Table 1: *NMR data of Rowithocin A (1) in methanol-d$_4$*

Pos.	δ_C	m	δ_H	m (*J* [Hz])	COSY	NOESY	H in HMBC
1	172.80	C				-	2ab, 23
2a	40.63	CH_2	2.99	br d (15.1)	2b, 31b >31a	(2b)> 5b, 31b >7, 32	31ab, 4
2b			2.88	dt (15.1, 1.7)	2a, 31ab	(2a)> 32, 4, 31b >6	
3	148.14	C	-		-	-	32, 2ab, 5ab, 4, 31ab
31a	111.35	CH_2	4.91	under OH signal	31b, 2b >2a	(31b) 4, 32 >5b >5a	2ab, 4
31b			4.78	br s	31a, 2ab	(31a) >2ab >22b	
4	42.06	CH	2.45	dqd (10.9, 6.9, 4.5)	32, 5b >5a	31a, (32) >2b, 6 >7	32, 5ab, 2ab, 31ab
32	20.99	CH_3	1.12	d (6.9)	4	(4) >5a, 31a, 2b, 5b >6	5b, 4
5a	41.28	CH_2	2.39	br dt (13.4, 4.5)	5b, 4, 6 >7	(5b) >32 > (6), (>7)	32, 7, 6
5b			2.13	dt (13.5, 10.9)	5a, 4, 6	(5a) >2a, 7 >32, 31a	
6	134.43	CH	5.63	ddd (15.1, 10.8, 4.7)	5ab, 7	8 >4, (5a) >2b, (5b)	8, 5ab
7	129.04	CH	6.57	br dd (15.2, 11.4)	6, 8 >5a	10, 5b > 2a >4, 5a	5b, 9
8	131.31[1]	CH	5.99	br t (11.2)	7, 9	6 (>9) >23	6, 10
9	129.52	CH	6.10	t (11.0)	8, 10	11, (8 >10) >19, 22a	11
10	130.62	CH	6.83	dd (15.1, 11.2)	9, 11	12, 7 (>9) >24, 15	8, 12
11	131.91	CH	6.77	dd (15.1, 11.0)	10, 12	9, 15, 7 >17 >33	13, 9
12	131.31	CH	6.25	t (10.9)	11, 13	10, (13) >26 >24	10
13	135.91	CH	6.01	br d (11.8)	33, 12 >15	33, (12) >15	33, 15, 11
14	138.44	C	-	-	-	-	33, 16, 12
33	17.72	CH_3	1.88	m	13, 15	16, 13 >11 >17	15, 13
15	129.78	CH	5.44	dquin (9.3, 1.3)	33, 16 >13	11, 19, 17 >13 >18 >26, 33, 35	33, 16, 13
16	72.00[2]	CH	4.88	br dd (9.3, 1.7)	15 >17	33 (>17) >18	15, 18, 19
17	81.90[3]	CH	4.71	br ddd (8.5[4], 8.0, 1.7)	18 >16	19 (>18), 11	19, 16, 15
18	128.72[5]	CH	5.89	dd (15.9, 8.0)	17, 19	34 >(>17) >16, 15	17, 16
19	130.20	CH	6.49	d (15.9)	18	22a, 17, 15 >11, 9	17, 34
20	135.06	C		-	-	-	34, 18, 19, 22a
34	21.30	CH_3	1.85	m	21, 22b	21, 18 >35, 9	19, 21
21	126.52	CH	5.20	br dd (11.4, 4.5)	34, 22ab	34 >(22b) >25	34, 22a, 19
22a	33.84	CH_2	2.56	dt (14.3, 10.0)	22b, 23, 21	(22b) >19, 24, (23), (21) >9, 8	23, 24
22b			2.11	br m	22a, 23, 21, 34	(22a) >(23, 21) >31b >24	
23	76.43	CH	5.13	ddd (9.3, 8.2, 3.4)	22ab, 24	25, (22b >22a) >31b	22a, 25, 24 >34
24	127.61	CH	5.26	ddd (15.5, 8.0, 1.1)	23, 25	26 >22a >35 >10, 12	22a, 26
25	140.11	CH	5.53	dd (15.3, 7.3)	26, 24	23 (>26), 35	35, 26, 23, 27
26	36.61	CH	3.30	ddq (9.0, 7.4, 6.9)	35, 27, 25	(35) >24, 29 >(25), 27, 15, 12, 13	35, 24, 25, 28
35	21.78	CH_3	0.99	d (6.9)	26	(26) >>27 >25 >15, 24, 34 >12, 13, 18	26, 25, 27
27	136.94	CH	5.23	m[6]	26, 28	>35 (28/30a)	35, 26, 25, 29
28	129.46	CH	5.96	tq (10.9, 0.9)	27, 29	30a/27	26, 30ab, 29
29	133.78	CH	6.68	dddd (16.9, 11.0, 10.2, 1.1)	30ab, 28	26 (>30b)	27
30a	117.98	CH_2	5.22	br d (15.9)[7]	29	(30b), 28	28
30b			5.16	dt (10.2, 2.1)	29	(30a, 29)	

[1] C-8 and C-12 overlap
[2] d, J = 4.5 Hz ^{31}P,^{13}C;
[3] d, J = 5 Hz ^{31}P,^{13}C
[4] J = 8.5 Hz ^{31}P,^{1}H coupling
[5] d, J = 5 Hz ^{31}P,^{13}C
[6] overlapping with H-30a
[7] from *J*res-spectrum

Supplementary Table 2: *Vicinal coupling constants (J) observed in rowithocin (1) and corresponding angles in the calculated model.*

H-#	H-#	*J* Hz]	Angle	H-#	H-#	*J* Hz]	Angle
4	5a	4.5	68	17	18	8.0	157
4	5b	10.9	-176	21	22a	9.3	-170
5a	6	4.5	-71	21	22b	4.5	-54
5b	6	10.9	173	22a	23	9.3	-157
7	8	11.3	-175	22b	23	3.4	87
9	10	11.2	173	23	24	8.1	-176
11	12	11.0	169	25	26	7.3	176
15	16	9.3	-179	26	27	9.0	-171
16	17	1.5	87				

Supplementary Table 3: *Intensities of $^1H,^1H$ ROESY correlation signals and distances in the rowithocin model.*

H-#	H-#[a]	NOE[b]	Dist. [Å]	H-#	H-#[a]	NOE[b]	Dist. [Å]
2a	4	+	3.67	13	15	+	3.46
	5b	++++	1.79		26	+	3.13
	7	++	2.89		33	+++	2.51
	32	+	2.81	15	17	++	2.78
	31b	++	3.75		18	+	3.84
2b	4	+	3.98		19	+++	2.58
	5a	+	4.50		22a	--	3.53
	31b	+	3.21		26	++	2.45
	32	+++	2.54		35	+	2.50
31a	32	+	3.38	16	18	+	3.84
	4	+++	2.32		33	+++	2.37
4	5a	-[c]	2.54	17	18	++	2.72
	6	+++	2.50		19	+++	2.44
5a	6	+++	2.61	18	34	+++	2.46
	7	+	3.36	19	22a	++++	1.78
	32	+++	2.45		22b	--	3.33
5b	7	+++	2.36	21	22a	+[d]	3.11
	32	++	2.60		22b	+++[d]	2.48
6	8	+++	2.49		23	-[e]	2.51
7	10	++++	1.80		25	+	4.00
8	22a	+	3.76		34	+++	2.30
9	11	+++	2.46	22a	23	++[d]	3.09
	19	+	3.16		24	++	2.49
	22a	+	3.04	22b	23	+++[d]	2.72
10	12	+++	2.46		31b	+	3.61
	15	+	4.51	23	31b	+	3.75
11	15	+++	2.55		25	+++	2.38
	17	++	2.62	24	26	+++	2.38
	19	+	2.90		35	+	3.80
	22a	-	3.50	25	26	++	3.13
	33	+	3.62		35	++	2.57
12	13	+++	2.43	26	29	++++	1.79
	15	+	3.85	27	35	+++	2.58
	26	+	3.51	28	30a	+++	2.49
				29	30b	++	2.48
				34	35	+	4.10

[a] only correlations with protons of higher numbering are given in the second column; [b] ++++ very strong, +++ strong, ++ medium, + low intensity; [c] only coupling visible; [d] with vicinal coupling; [e] overlap with 30a/30b;

Supplementary Table 4: *In vitro antibacterial and antifungal activity of rowithocin (1) dissolved in acetonitrile, and three control drugs as reference. 20 µL of MeOH and acetonitrile without drugs, respectively, showed no effect on tested organisms. MIC columns show the determined Minimum Inhibitory Concentrations (µg/mL).*

Test organism	DSMZ No.	MIC (rowithocin) [µg/mL]	MIC (reference) [µg/mL]
Mucor hiemalis	2656	n.i.	4.2 [c]
Candida albicans	1665	n.i.	8.3 [c]
Wickerhamomyces anomalus	6766	n.i.	16.6 [c]
Rhodotorula glutinis	10134	n.i.	2.1 [c]
Schizosaccharomyces pombe	70572	n.i.	16.6 [c]
Micrococcus luteus	1790	n.i.	0.42 [a]
Bacillus subtilis	10	n.i.	8.3 [a]
Staphylococcus aureus	346	3.3 - 4.2	0.21 [a]
Mycobacterium sp.	43270	n.i.	>33.3 [a]
Chromobacterium violaceum	30191	16.6	0.83 [a]
Escherichia coli	1116	67.0	0.83 [a]
Pseudomonas aeruginosa	50071	n.i.	2.1 [b]
Mouse fibroblast cell line L929 (IC_{50})		22 µg/mL	

[a] Oxytetracyclin hydrochloride; [b] Gentamycin; [c] Nystatin, n.i.: no inhibition. The cell density was adjusted to 8×10^6 cells/mL. *spores from agar-plate were applied without justification; DSMZ: German Collection of Microorganisms and Cell Cultures Braunschweig

Supplementary Table 5: *Representative readouts from hypothesis testing (paired t-tests) on species, genus, family, suborder level.*

Species								
Pair	Column	N	Missing Count	Mean	Standard Deviation	Standard Error Mean		
Pair 1	Distance	3720	0	0.649830234624685	0.16385923956657006	0.0026865780684917195		
Pair 1	Distance (#1)	3720	0	0.5806919869726337	0.14972239922459613	0.0024547954401762354		
Pair	Label	t	df	p-value (2-tailed)	Mean	Standard Deviation	Standard Error Mean	
Pair 1	Distance - Distance (#1)	1,999E+16	3719	1,71E-68	0.06913824765205119	0.210990327083478	0.003459322690046289	
Confidence Interval Probability		Confidence Interval of the Difference (Lower Bound)			Confidence Interval of the Difference (Upper Bound)			
0.99		0.06022304743395297			0.07805344787014941			
Genus								
Pair	Column	N	Missing Count	Mean	Standard Deviation	Standard Error Mean		
Pair 1	Distance	11555	0	0.8238539193986807	0.1143885499676932	0.0010641370881724701		
Pair 1	Distance (#1)	11555	0	0.6169423579651767	0.15305990576480738	0.0014238900876225474		
Pair	Label	t	df	p-value (2-tailed)	Mean	Standard Deviation	Standard Error Mean	
Pair 1	Distance - Distance (#1)	1,181E+16	11554	0.0	0.20691156143350087	0.18839489957608438	0.0017526054829618914	
Confidence Interval Probability		Confidence Interval of the Difference (Lower Bound)			Confidence Interval of the Difference (Upper Bound)			
0.99		0.20239640297466177			0.21142671989233996			
Family								
Pair	Column	N	Missing Count	Mean	Standard Deviation	Standard Error Mean		
Pair 1	Distance	23616	0	0.8474435252600174	0.11011123501427696	7,17E+10		
Pair 1	Distance (#1)	23616	0	0.727467669479465	0.14854696542146004	9,67E+11		
Pair	Label	t	df	p-value (2-tailed)	Mean	Standard Deviation	Standard Error Mean	
Pair 1	Distance - Distance (#1)	1,009E+16	23615	0.0	0.11997585578055697	0.18265775828001046	0.0011885978853637049	
Confidence Interval Probability		Confidence Interval of the Difference (Lower Bound)			Confidence Interval of the Difference (Upper Bound)			
0.99		0.1169139830371939			0.12303772852392005			
Suborder								
Pair	Column	N	Missing Count	Mean	Standard Deviation	Standard Error Mean		
Pair 1	Distance	61855	0	0.8358481898506264	0.10832587519549831	4,36E+12		
Pair 1	Distance (#1)	61855	0	0.7727541041562909	0.14735899306709876	5,93E+11		
Pair	Label	t	df	p-value (2-tailed)	Mean	Standard Deviation	Standard Error Mean	
Pair 1	Distance - Distance (#1)	8,712E+15	61854	0.0	0.06309408569433975	0.1801183633016879	7,24E+11	
Confidence Interval Probability		Confidence Interval of the Difference (Lower Bound)			Confidence Interval of the Difference (Upper Bound)			
0.99		0.06122856071846816			0.06495961067021133			

Supplementary Table 6: *Cultivation media recipes. All media were prepared using MilliQ water. Sterilization was done by steam-autoclaving.*

Medium	Recipe		Medium	Recipe	
A	4 g/L	glycerol	H	2 g/L	soy flour
	8 g/L	starch		2 g/L	glucose
	4 g/L	soy flour		8 g/L	starch
	2 g/L	yeast extract		2 g/L	yeast extract
	1 g/L	$CaCl_2$*2H_2O		1 g/L	$CaCl_2$*2H_2O
	1 g/L	$MgSO_4$*7H_2O		1 g/L	$MgSO_4$*7H_2O
	11.9 g/L	HEPES (50 mM)		11.9 g/L	HEPES (50 mM)
	8 mg/L	Fe-EDTA		8 mg/L	Fe-EDTA
	pH 7.4 with 10 N KOH			pH 7.4 with 10 N KOH	
CLF	4 g/L	fructose	P	2 g/L	peptone
	6 g/L	glucose		8 g/L	starch
	10 g/L	skim milk powder		4 g/L	probion
	2 g/L	yeast extract		2 g/L	yeast extract
	1 g/L	$CaCl_2$*2H_2O		1 g/L	$CaCl_2$*2H_2O
	1 g/L	$MgSO_4$*7H_2O		1 g/L	$MgSO_4$*7H_2O
	11.9 g/L	HEPES (50 mM)		11.9 g/L	HEPES (50 mM)
	8 mg/l	Fe-EDTA		8 mg/L	Fe-EDTA
	pH 7.0 with 10 N KOH			pH 7.5 with 10 N KOH	
CTT	10 g/L	casiton	Pol3	3 g/L	probion
	1 mL/L	1 M K2HPO4 in H_2O		3 g/L	starch
	2 g/L	MgSO4*7H_2O		0.5 g/L	$CaCl_2$*2H_2O
	1.21 g/L	TRIS (10 mM)		2 g/L	$MgSO_4$*7H_2O
	pH 7.6 with 1 M HCl			11.9 g/L	HEPES (50 mM)
				pH 7.2 with 10 N KOH	
Cy	3 g/L	casiton	S	4 g/L	soy flour
	1 g/L	yeast Extrakt		2 g/L	glucose
	1 g/L	$CaCl_2$*2H_2O		8 g/L	starch
	11.9 g/L	HEPES (50 mM)		1 g/L	$CaCl_2$*2H_2O
	pH 7.2 with 10 N KOH			1 g/L	$MgSO_4$*7H_2O
				11.9 g/L	HEPES (50 mM)
				8 mg/L	Fe-EDTA
				pH 7.4 with 10 N KOH	
E	5 g/l	glycerol			
	4 g/L	skim milk powder			
	4 g/L	soy flour			
	2 g/L	yeast extrat			
	10 g/L	starch			
	1 g/L	$MgSO_4$*7H_2O			
	11.9 g/L	HEPES (50 mM)			
	8 mg/l	FeEDTA			
	pH 7.4 with 10 N KOH				

Supplementary Table 7: *Blank feature lists used for matrix feature filtering*

Blank List Name	Blank data sets from various cultivation media	Used for	# features
01_blankfeatures_A-CTT	A, CTT	*Myxococcus, Nannocystis*	4780
02_blankfeatures_A-CTT-Pol3	A, CTT, Pol3	*Chondromyces*	5291
03_blankfeatures_H-P	H, P	*Corallococcus, Sorangium*	7931
04_blankfeatures_CTT-H-P-Pol3	CTT, H, P, Pol3	*Cystobacter, Stigmatella*	10780
05_blankfeatures_CTT-Pol3	CTT, Pol3	*Polyangium*	2985

Supplementary Table 8: *Taxonomic distribution of strains and their count, according to Figure 1 and Figure 2 of the main text (total count: 2316).*

Aetherobacter	fasciculatus	1
Aetherobacter	rufus	1
Aetherobacter	unclassified	3
Archangium	gephyra	13
Archangium	unclassified	12
Archangium	violaceum	1
Atactangium	chaos	2
Byssovorax	cruenta	1
Chondromyces	apiculatus	12
Chondromyces	catenulatus	1
Chondromyces	crocatus	11
Chondromyces	lanuginosus	2
Chondromyces	pediculatus	16
Chondromyces	robustus	7
Chondromyces	unclassified	1
Corallococcus	coralloides	104
Corallococcus	exiguus	31
Corallococcus	macrosporus	16
Corallococcus	unclassified	98
Cystobacter	armeniaca	13
Cystobacter	badius	14
Cystobacter	disciformis	40
Cystobacter	ferrugineus	24
Cystobacter	fuscus	24
Cystobacter	gracilis	21
Cystobacter	miniatus	3
Cystobacter	minor	6
Cystobacter	minus	2
Cystobacter	unclassified	23
Cystobacter	velatus	27
Cystobacter	violaceus	45
Hyalangium	minutum	19
Hyalangium	unclassified	3
Jahnella	thaxteri	6
Jahnella	unclassified	2
Kofleria	flava	7
Melittangium	boletus	9
Melittangium	lichenicola	34
Melittangium	unclassified	1
Minicystis	unclassified	2
Myxococcus	fulvus	259
Myxococcus	macrosporus	2
Myxococcus	stipitatus	76
Myxococcus	unclassified	116
Myxococcus	virescens	191
Myxococcus	xanthus	155
Nannocystis	exedens	117
Nannocystis	pusilla	13
Nannocystis	unclassified	16
Phaselicystis	flava	1
Polyangium	fumosum	7
Polyangium	sorediatum	7
Polyangium	spumosum	12
Polyangium	unclassified	86
Pyxidicoccus	fallax	5
Pyxidicoccus	unclassified	18
Sandaracinus	amylolyticus	1
Sorangium	cellulosum	451
Sorangium	compositum	1
Sorangium	nigrum	37
Sorangium	unclassified	10
Stigmatella	aurantiaca	21
Stigmatella	erecta	44
Stigmatella	hybrida	12

Supplementary Note 1

Identification of Rowithocin, a novel Natural Product from *Sorangium cellulosum*

Rowithocin is a compound which undergoes degradation within days. Consequently, rowithocin was detected in extracts mostly in the form of more stable elimination products, the most prominent one being represented by bucket #55784 (16.80 min, 497.339 *m/z*; Figure 5 of main text). The mass spectra indicated a strong in-source fragmentation of the compound, based upon which extracted ion chromatograms of those *m/z* values pointed on tentative additional rowithocin derivatives. Only measurements of freshly prepared extracts allowed us to identify the phosphorylated rowithocin (as displayed in Supplementary Fig. 2), which is to our understanding the product of biosynthesis and represented by MS signals within the red area in Supplementary Figure 1 (see next paragraph for an explanation of MS signals). An UV absorption with a maximum at 320 nm was another strong indicator for the different rowithocin derivatives in this extract. See Supplementary Table 4 for bioactivity measures.

Structure Elucidation

HRESIMS of **1** in the negative mode showed a prominent $[M\text{-}H]^-$ signal at 609.2987 *m/z* while in positive ESI mode only a very low abundant $[M+H]^+$ at *m/z* 611.3119 was observed. Both clusters suggested the elemental composition $C_{35}H_{47}O_7P$ for rowithocin (**1**) which was supported by a sodium adduct ion cluster $[M+Na]^+$ as well as a collection of additional neutral loss-derived signals. The most abundant ions in positive mode were fragments at 513.3361 *m/z* and 495.3255 *m/z*, for which calculated molecular formulas fit to $[M–H_3PO_4+H]^+$ and $[M–H_3PO_4\text{-}H_2O+H]^+$ ion clusters and thus established **1** as the phosphoric ester.

The NMR spectra of **1** in methanol-d_4 were well resolved and all protons visible in the ^{1}H NMR spectrum could be correlated to their corresponding carbons from the ^{1}H,^{13}C-HSQC-DEPT spectrum, leaving only 3 unsaturated quaternary carbon atoms and a carboxylic carbon (C-1) at δ_C 172.8 ppm (Supplementary Table 1).

The ^{1}H,^{1}H COSY spectrum contained correlation sequences of three large building units, i.e. unit A from methyl group C-32 to the unsaturated methine C-13, unit B from C-15 to C-19, and unit C from C-21 to C-30. Units A and B were connected via the unsaturated quaternary carbon C-14 showing ^{1}H,^{13}C HMBC

correlations with methyl group C-33 and methines C-16 and C-12 and from mutual correlations of methines C-13 and C-15. Similarly, the unsaturated quaternary carbon C-20 bearing methyl group C-34 was identified as connection between units B and C (Supplementary Table 1).

The only remaining correlations in the COSY spectrum indicated long-range couplings between an unsaturated exocyclic methylene group (C-31) and methylene group C-2. These were supported by mutual $^{1}H,^{13}C$ HMBC correlations. Their $^{1}H,^{13}C$ HMBC correlations with the unsaturated quaternary C-3 completed this small structure element. Further HMBC correlations of these four methylene protons with methine C-4 and correlations of C-3 with H-4, methyl protons at C-32 and methylene protons at C-5 designated the connection of C-3 to structure element A. Finally, the HMBC correlations of the methylene protons at C-2 with the carboxyl group C-1 completed the carbon skeleton of **1**. The oxymethine group C-23 with the highest proton shift (δ_H = 5.13 ppm) suggested the position of the lactone ring closure which was additionally supported by the HMBC correlation of H-23 with the carboxyl group C-1.

The ^{1}H NMR spectrum of **1** in methanol-d_4 was well resolved at 700 MHz and allowed the assignment of all vicinal coupling constants at least from one of the coupling protons. Consequently, *trans* configurations of the $\Delta^{6,7}\Delta$,$^{10,11}\Delta$,^{18,19}, and $\Delta^{24,25}$ double bonds were derived from their vicinal coupling constants between 15.1 and 15.9 Hz while values around 11 Hz characterized the *cis* configurations of the $\Delta^{8,9}\Delta$,^{12,13}, and $\Delta^{27,28}$ double bonds. The cis configuration of the methyl substituted $\Delta^{20,21}$ double bond was recognized from strong ROESY correlations of methyl group C-34 with 18-H and 21-H and a correlation between H-19 and Ha-22 while the *trans* configuration of the $\Delta^{14,15}$ double bond was shown by ROESY correlations of methyl group C-33 with H-16. Another strong ROESY correlation between methyl group C-33 and methine H-13 and a ROESY correlation between H-11 and H-15 indicated a *s-cisoidal* conformation between the $\Delta^{12,13}$ and $\Delta^{14,15}$ double bonds, which presumably was enforced by the ring strain of the lactone. The ring strain is caused by the presence of a pentaene, a diene, an exomethylene and the lactone group in the 24-membered ring. Together with the two *cis* double bonds this spatial arrangement caused the unexpected low UV maximum at 320 nm of the pentaene chromophore.

Beyond the double bond configurations the vicinal coupling constants over single bonds clearly suggested the spatial arrangements of methine and methylene protons (Supplementary Table 2) especially in the lactone ring, i.e. fully staggered conformations were recognized from *J*-values between

9.3 and 10.9 Hz for Hb-5 with H-6 and H-4 as well as for 22a-H with 21-H and H-23. Similarly the large coupling constants of 9.3 and 8.0 Hz indicated fully staggered or nearly fully staggered orientations of H-15 and H-16 as well as H-17 and H-18, respectively. On the other hand a conspicuously small *J*-value of 1.5 Hz between H-16 and H-17 suggested an angle of about 90 degree between these protons.

These unambiguous indications of a well-defined conformation of the lactone ring triggered the experiment of calculating an appropriate conformation or relative configuration of rowithocin (**1**) which additionally should explain/account for the observed NOE correlations. Initially, the bare ring containing all double bonds and their methyl groups and the exomethylene group was optimized using the "Conformational Search" module of HyperChem Professional Ver. 8.0 with the mm+ force field. The conformation was then refined by stepwise substitution of protons by a methyl group at C-4 and by hydroxyl groups at C-16 and C-17, by a phosphate group at C-17 and by the side chain at C-23. The methyl branch of the side chain was calculated as S and R conformation. After each step the torsion angles of the optimized conformers were checked to roughly match the vicinal coupling constants and the main long-range NOEs. Finally, the force-field generated conformer was optimized using the semi-empirical pm3 method (Supplementary Fig. 4). Analysis of the torsion angles provided the values collected in Supplementary Table 2, which were in due proportion to the vicinal coupling constants observed. All large coupling constants of about 8 – 11.3 Hz were related to torsion angles between 157 and 179 degree, and the small coupling of 1.5 Hz observed for $^3J_{16,17}$ matched the torsion angle of 87 degree in the model of **1** according to the Karplus curve with the broad minimum at about 90 degree. Further the intensities of the ROESY signals were compared to the distances in the model. All small distances under 2 Å were represented in the ROESY spectrum by very strong correlation signals. Strong correlation signals were found for distances up to about 2.6 Å while weak signals were observed for distances over 2.8 Å. However, although the distances of H-15 to H-26 and to methyl group C-35 were 2.45 and 2.5 Å, respectively, the ROESY correlation signals were assessed smaller than expected. This observation probably is due to the motility of the side chain in relation to the lactone ring, which will be supported by the solvent molecules.

General Experimental Procedures

UV data were recorded on a Shimadzu UV/vis-2450 spectrophotometer in methanol (UVASOL, Merck); ^1H, ^{13}C, and ^{15}N NMR spectra were recorded on Bruker AVANCE III HD 700 MHz with Cryo Platform or Bruker Avance III 500 MHz spectrometers, locked to the deuterium signal of the solvent. Data

acquisition, processing and spectral analysis were performed with standard Bruker software and ACD/Spectrus Processor. Chemical shifts are given in parts per million (ppm) and coupling constants in Hertz (Hz). HRESIMS data were recorded on a Maxis ESI TOF mass spectrometer (Bruker Daltonics), molecular formulas were calculated including the isotopic pattern (Smart Formula algorithm). Analytical RP HPLC was carried out with an Agilent 1260 HPLC system equipped with a diode-array UV detector (DAD) and a Corona Ultra detector (Dionex) or a Maxis ESI TOF mass spectrometer (Bruker Daltonics). HPLC conditions: Waters Acquity C_{18} column 50×2.1 mm, 1.7 µm dp, solvent A: H_2O, 0.1% HCOOH; solvent B: acetonitrile, 0.1% HCOOH; gradient system: 5% B for 1 min, increasing to 95% B in 20 min; flow rate 0.6 mL/min; 40 ^oC.

Extraction and Isolation of Rowithocin

90 mL of adsorber resin XAD-16 from a 9.5 L culture of strain *S. cellulosum* MSr2476 was separated from cell mass by floating and extracted in a glass column using 400 mL of methanol. The organic solvents of the eluate were evaporated to obtain an aqueous mixture. It was diluted with water to give about 170 mL before being extracted three times with ethyl acetate (170 mL each). The combined organic layer was evaporated to a thin syrup, which was immediately re-dissolved in 95% methanol with 5% water (200 ml) and subjected to solvent-solvent partitioning with n-heptane (200 mL). The partitioning was repeated thrice and removed 274 mg lipophilic compounds with the heptane layers. The methanol was evaporated and the remaining aqueous mixture was diluted with 90 mL of water and extracted thrice with 100 mL of dichloromethane to give 644 mg of crude product. Separation of this material on Sephadex LH-20 [column 5 x 90 cm using methanol (flow 7.8 mL/min), detection UV at 313 nm) furnished two main fractions both containing rowithocin A (Fraction 1: 150 – 184 min, 152 mg; Fraction 2: 184 – 216 min, 35 mg).

Fraction 1 was further separated into four portions by RP-HPLC [Nucleodur 100-10 C_{18} EC column; 250x21 mm; solvent A: 50% aqueous methanol; solvent B: 100% methanol; each with 50 mMol NH_4Ac and 1 mL/L of acetic acid; gradient 55% B for 30 min., increasing to 100% B in 20 min.; flow 20 mL/min.; UV detection at 310 nm] to give a main peak at ~32 min.. The organic solvent was removed by evaporation and the remaining buffer was extracted with ethyl acetate. After evaporation to dryness 7.7 mg of an enriched rowithocin extract were obtained and further purified by RP-HPLC under the same conditions, however with solvent A:B = 1:1 [solvent A: acetonitrile/water 5:95 (v/v) with 50 mMol NH_4Ac and 0.4 mL/L of acetic acid; solvent B: acetonitrile/water 95:5 (v/v) with 10 mMol NH_4Ac and 0.08 mL/L of acetic acid] to give 2.7 mg of pure rowithocin at 11.6 min..

Fraction 2 was separated by RP-HPLC [Nucleodur 100-10 C_{18}EC column; 250x21 mm; solvent A: 50% aqueous methanol; solvent B: 100% methanol; each with 50 mMol NH_4Ac and 1 mL/L of acetic acid; gradient 55% B to 100% B in 60 min.; flow 20 mL/min.; UV detection at 310 nm] to give further 3 mg of rowithocin.

Rowithocin (**1**): $C_{35}H_{47}O_7P$, M = 610.71; $[\alpha]^D_{22}$ = - 72.7 (*c* = 0.0172, MeOH); HPLC system R_t = 14.4 min; UV (MeOH) λ_{max} (log ε) 233 (4.460), 320 (4.267) nm; NMR data, see table; (+)-HRESIMS *m/z* 495.3255 [M-H_3PO_4-H_2O+H]$^+$ (calcd for $C_{35}H_{43}O_2$, 495.3257), *m/z* 513.3361 [M-H_3PO_4+H]$^+$ (calcd for $C_{35}H_{45}O_3$, 513.3363), *m/z* 611.3119 [M+H]$^+$ (calcd for $C_{35}H_{48}O_7P$, 611.3132), *m/z* 633.2948 [M+Na]$^+$ (calcd for $C_{35}H_{47}O_7P$+Na, 633.2952); (-)-HRESIMS *m/z* 609.2987 [M-H]$^-$ (calcd for $C_{35}H_{46}O_7P$, 609.2986).

Supplementary Note 2

Data Analysis Workflow

At the core of this project is the untargeted analysis of mass spectrometric features. The general data analysis procedure is illustrated in Supplementary Fig. 5. The required functions were implemented using a combination of the KNIME analytics platform, the R statistics package, and custom modules programmed in Java and executed server-side within the Apache Thrift remote procedure framework. Data handling throughout the project was supported by a MySQL database system, operated as part of the in-house research database, Myxobase.

The developed software modules including source code are publicly available in a repository located at https://bitbucket.org/hipsprojects/.

Sampling size Effects

To check how many representatives of each genus are necessary to maintain the clustering of the data a randomized sampling of 20 representatives for seven major genera was done. After clustering the datasets based on their known secondary metabolite profile, the number of representatives was reduced to half by randomly removing data sets. The same clustering approach was repeated with the smaller sample set and the resulting hierarchical tree color-coded according to the different genera (Supplementary Fig. 6). General trends in clustering remain intact even for the reduced dataset. We conclude that when using the known secondary metabolite profiles, sampling of only 10 of each genus is

already sufficient for a reasonable clustering. The reason is that many genera have very characteristic compounds that are unique to their genus and as such allow a straightforward allocation in the clustering approach.

Media Effects on Clustering

Most *Polyangium* and *Chondromyces* from the suborder *Sorangiineae* are grown in the same medium (Pol3), and thus the theoretical possibility exists that clustering occurs as a consequence of media-derived MS signals. Although the implemented background filtering procedure should mitigate this possibility, we sought to elaborate further on this point. A suitable experiment became apparent when we analyzed all 499 available *Sorangium* data sets of our repository. Those were mainly grown in two similar media, with or without yeast extract, H and S medium, respectively. The analysis was again solely based on mass spectral features which were filtered for polymer and media-related blank features. The distribution matrix derived from the bucketing process had 7000 buckets which were eventually used for hierarchical clustering as described in the online methods section. Thereafter, the ordered data sets were colored according to the medium that was used for extract preparation to visualize any clustering effects (Supplementary Fig. 7).

By recognizing that even the absence or presence of yeast extract had no obvious influence on clustering we reasoned that the clustering is only scarcely affected by the media features. While a minimal residual influence cannot be excluded with certainty, our results indicate that this effect is not relevant for the present study as each genus features its own characteristic pattern in the heatmap.

Additional Considerations

Clustering solely based on *identified* known compounds could easily lead to wrong conclusions whenever the sample size is too low and certain compound families appear in different genera. A case scenario would be that a strain of genus B lacks secondary metabolites that are typical for its genus but features a metabolite typical for genus A. Consequently, the B strain will be allocated to the A clade. One such example is shown below with the natural product apicularen. As a reminder, 50 representatives of each genus were randomly chosen from the complete list of available data sets. Clustering and heatmapping the data resulted in a *Chondromyces* clade of apicularen producers. Within this clade a single *S. cellulosum* member (s_MXID 1158) is found (highlighted in red in Supplementary Fig. 8).

Double-checking this result using our database proves that apicularen is mainly produced by *Chondromyces* although a small number of *Sorangium* sp. are known to produce it as well. The producers are listed below with a total of 11 different *Sorangium* producers out of 499 *Sorangium* data sets.

#	s_MXID	Genus	Species
1	1543	Sorangium	cellulosum
2	1156	Sorangium	cellulosum
3	1760	Sorangium	cellulosum
4	2470	Sorangium	cellulosum
5	1769	Sorangium	cellulosum
6	5803	Sorangium	cellulosum
7	6538	Sorangium	cellulosum
8	63	Sorangium	cellulosum
9	1158	Sorangium	cellulosum
10	2197	Sorangium	cellulosum
11	1795	Sorangium	cellulosum

The fact that the used dataset had almost only *Chondromyces* producers of apicularen resulted in the grouping of the *Sorangium cellulosum* amongst the *Chondromyces*. In this scenario we knew this is a *Sorangium* species. Assuming we would want to assign this extract to a genus simply based on the known-compound fingerprint, one would certainly end up with a misclassification of this particular *Sorangium* as *Chondromyces*.

Consequently, any classification attempts just based on *known secondary metabolite* profiles is not feasible as it easily causes false classifications. In the above shown scenario, one reason for the misclassification is related to the low sample size with respect to apicularen producing *Sorangium* species. Also, the number of known compounds within each dataset varies dramatically. Strains with only a few or a single known compound are plausibly prone to incorrect clustering. Clustering based on the comprehensive secondary metabolite profile –including unknowns - is thus regarded superior over focusing on only the known metabolites.

In this study we focus on HPLC/MS-detectable metabolites occurring with a non-ubiquitous pattern as a consequence of evolutionary diversification, comprising the subset of products from a variety of secondary metabolite biosynthetic pathways (the secondary metabolome). Therefore, although we cannot exclude *per se* the possibility, the specific analysis scenario makes it less likely that significant numbers of molecules originating from primary metabolic processes (which are fundamentally the same within the bacterial order) are identified. In addition, the extraction procedure as well as analytical parameters (RP-HPLC conditions, mass range) discriminate against molecules with properties shared by many primary metabolites, e.g. highly hydrophilic compounds in the low molecular mass range.

Clustering analysis to highlight unknowns

Figure 4 in the main text presents a clustering analysis of 515 datasets, the composition of which results from the following procedure: 1. selecting 50 random datasets from all genera where more than 50 are available and 2. adding all datasets from the genera where less than 50 are available. Supplementary Fig. 9 shows results from two additional analysis runs, confirming that the overall picture when using new random-sample pools remains structurally stable, i.e. revealing clusters of genus-specific buckets.

For an overview of unique or highly specific features revealed by taxonomy-resolved clustering, the underlying features (in total 786) were visualized across the chromatographic retention time range and mass range used for LC-MS analysis, to give a plausible distribution in light of typical properties of known myxobacterial natural products such as molecular mass distribution often ranging from 400 – 800 m/z (Supplementary Fig. 10).

Profile similarity statistics

As part of this study, we analyzed the similarity of metabolite profiles as a function of phylogenetic relatedness, on the basis of metabolite distributions from 515 strains spanning 12 myxobacterial genera (main text Figure 5). For that purpose the occurrence pattern of 9247 compound buckets were converted into a matrix comprising 515 binary profiles and pairwise distance was determined using the bitvector cosine distance measure. We find that accumulated between-clade metabolite profile distances are on average larger than distance values calculated internally within members of the respective clade. To underline this result, Supplementary Fig. 11 presents pairwise graphs showing clade-internal distributions (blue) compared to between-clade distributions (green, red, violet, grey). Apart from the clearly visible shift, the means of similarity distributions were also compared using paired t-tests with a 99% confidence interval. Random subsampling was used to balance test group sizes, as numbers of distance pairs are inevitably unequal when calculated within/outside of clades. Hypothesis testing gives p-values close to zero for all four analyzed levels (representative readouts summarized below in Supplementary Table 5). Note that only four myxobacterial families are present in the sample set due to restricted availability of isolates, covering the three myxobacterial suborders. Due to this disproportion the explanatory power of analysis on family level vs. suborder level is limited and was therefore deliberately not included in the main text Figure 5.

Supplementary Note 3

Cultivation Media

Various cultivation media were used for growing the myxobacterial genera in the course of the project.

Genus	Media
Chondromyces	A, Cy, P, Pol3, CTT
Corallococcus	A, Cy, E, H, P, Pol3, CTT
Cystobacter	A, CLF, Cy, H, P, Pol3
Myxococcus	A, Cy, H, CTT
Nannocystis	A, Cy, P, CTT
Polyangium	A, CLF, Cy, H, Pol3, P, CTT
Sorangium	H, P, Pol3, S
Stigmatella	A, Cy, H, P, Pol3

Many of those media are similar in a sense that they contain almost the same components but at different concentrations. Thus LC-MS chromatograms of such media extracts look similar as well, e.g. A, H, S, E, thereby indicating that we can expect similar blank-related MS features from similar media. They do, however, differ from other media groups like the casiton-based Cy and CTT. Consequently, media-related features can be very different for the different genera. We decided to do the media filtering with lists that are tailored to the various genera. Eventually, we created five blank feature lists (see Supplementary Table 7). The rationale for selection laid in the composition of the media recipes and their use for the different genera (Supplementary Table 6). The resulting lists can have up to 10,000 blank features which are ought to be filtered from the extract data sets at an early step of the workflow.
