# Supplementary material for: Correlating chemical diversity with taxonomic distance for discovery of natural products in myxobacteria
Source: Nat Commun. 2018 Feb 23;9:803. doi: 10.1038/s41467-018-03184-1 (PMC5824889; doi:10.1038/s41467-018-03184-1)
Supplement: Supplementary file 2 — Description of Additional Supplementary Files [file 41467_2018_3184_MOESM2_ESM.pdf]

Description of Additional Supplementary Files

File Name: Supplementary Data 1
Description: List of myxobacterial strains used for statistical analysis.
